# Cerebellar‐hippocampal processing in passive perception of visuospatial change: An ego‐ and allocentric axis?

**DOI:** 10.1002/hbm.24865

**Published:** 2019-11-15

**Authors:** Maximilian F. A. Hauser, Stefanie Heba, Tobias Schmidt‐Wilcke, Martin Tegenthoff, Denise Manahan‐Vaughan

**Affiliations:** ^1^ Department of Neurophysiology, Medical Faculty Ruhr University Bochum Bochum Germany; ^2^ International Graduate School of Neuroscience Ruhr University Bochum Bochum Germany; ^3^ Department of Neurology BG University Hospital Bergmannsheil Bochum Germany; ^4^ Department of Neurophysiology Heinrich‐Heine University of Düsseldorf Düsseldorf Germany

**Keywords:** allocentric, cerebellum, egocentric, hippocampus, human, perception, spatial

## Abstract

In addition to its role in visuospatial navigation and the generation of spatial representations, in recent years, the hippocampus has been proposed to support perceptual processes. This is especially the case where high‐resolution details, in the form of fine‐grained relationships between features such as angles between components of a visual scene, are involved. An unresolved question is how, in the visual domain, perspective‐changes are differentiated from allocentric changes to these perceived feature relationships, both of which may be argued to involve the hippocampus. We conducted functional magnetic resonance imaging of the brain response (corroborated through separate event‐related potential source‐localization) in a passive visuospatial oddball‐paradigm to examine to what extent the hippocampus and other brain regions process changes in perspective, or configuration of abstract, three‐dimensional structures. We observed activation of the left superior parietal cortex during perspective shifts, and right anterior hippocampus in configuration‐changes. Strikingly, we also found the cerebellum to differentiate between the two, in a way that appeared tightly coupled to hippocampal processing. These results point toward a relationship between the cerebellum and the hippocampus that occurs during perception of changes in visuospatial information that has previously only been reported with regard to visuospatial navigation.

## 1 INTRODUCTION

The hippocampus is well known for its role in declarative memory and spatial navigation. In recent years, however, an increasing number of studies specifically designed to rule out any mnemonic elements, have put the hippocampus in the spotlight of perceptual processes (for a review, see Lee, Yeung, & Barense, 2012). Initial studies showed that hippocampal patients exhibit particular impairments in purely perceptual discrimination tasks. For example, when discriminating between simultaneously shown scenes on the basis of subtle differences, hippocampal patients displayed impairments compared to controls (Lee et al., 2005). This deficit was further elaborated in an oddity judgment paradigm, where participants are asked to identify the different image from a set of four (Lee, Bussey, et al., 2005). When the stimuli consisted of rooms, patients with hippocampal lesions had trouble selecting the odd image when viewpoints differed, but not when viewpoints remained the same (Lee et al., 2005); a finding that was later corroborated by functional magnetic resonance imaging (fMRI) data (Barense, Henson, Lee, & Graham, 2010).

Another series of studies on hippocampal patients (Aly, Ranganath, & Yonelinas, 2013; Aly, Wansard, Segovia, & Yonelinas, 2014) found perceptual impairments in the discrimination between pictures on the basis of global, feature‐relational differences that are seen when an image is warped slightly. Patients were unimpaired, however, in the detection of discrete changes, such as the removal or addition of objects in a scene. The fact that the perceptual impairment was only found with regard to “strength‐based” perception suggests that the hippocampus is particularly important in the processing of high‐resolution bindings, such as fine‐grained relationships between perceptual features (Yonelinas, 2013).

It has been proposed that these perceptual tasks recruit the hippocampus in a way that is related to its role in navigation (Zeidman & Maguire, 2016; Zeidman, Mullally, & Maguire, 2015). However, in considering the findings that both, perspective changes in the oddity judgment task, as well as the processing of feature relations, appear to depend on hippocampal function, a central question is whether and how these predominantly ego‐ and allocentric perceptual differences are differentiated in the hippocampus and connected networks. Both, a shift of one's own relationship to a visual item, as well as a change in the item itself, can produce fine‐grained changes in visual feature‐relations, though they would each place very different demands on hippocampal representations.

This question is addressed here by means of fMRI and electroencephalography (EEG), where human subjects viewed three‐dimensional constructs passively in an oddball‐like manner: While subjects performed a simple distractor‐task, a standard variant of one such structure was repeatedly shown, until, after a minimum of seven repetitions, a changed variant of this structure was shown. These changes, all of which were equally unpredictable, consisted of the structures being shown in a new configuration, or from a different viewpoint.

These experimental manipulations, thus, both presented novel feature bindings at the perceptual level that could each be expected to recruit the hippocampus. In a spatial context, however, they arose in different frames of reference, and could thus be expected to effectuate different updating processes in the hippocampus. The changes in perspective effectively changed the relationship between the viewer and the structure, and can, thus, be seen as having more of an egocentric, or ego‐relative nature (Filimon, 2015). By contrast, configurational alterations happened in an object‐relative space, where we define “objects” as the different components making up the structures. These changes are, thus, more of an allocentric nature, though we wish to point out that this does not necessarily transfer to the map‐like representations believed to be involved in navigation.

We postulated that the differences between the conditions would be most visible at a network level that involves the parietal cortex as a central hub for reference frame conversions (Chafee, Averbeck, & Crowe, 2007; Crowe, Averbeck, & Chafee, 2008). We hypothesized that the superior parietal cortex is particularly tasked during changes to viewpoints, due to the role it is suggested to play in egocentric updating (Burgess, 2008). Comparable to the hippocampus' recent emergence as a perceptual structure, the medial posterior parietal cortex has recently been demonstrated to be involved in mnemonic functions. It is worth noting that at this level, the two structures behave in opposite ways: whereas the hippocampus responds to initial, novel experiences, the parietal cortex, and particularly the precuneus, is sensitive to repeated retrieval of information (Brodt et al., 2016; Brodt, Gais, Beck, Erb, & Scheffler, 2018; Gilmore, Nelson, & Mcdermott, 2015; Schott et al., 2019). On this basis, we hypothesized that insofar as a viewpoint change is correctly identified as a repeated, but also transformed presentation of the same structure, the medial posterior parietal cortex would be activated, whereas novel configurations of the structure would rather elicit hippocampal responses.

Our hypotheses held true with respect to the cortical responses to the viewpoint changes. In our fMRI‐data, we also found that the anterior hippocampus, strikingly in concert with the cerebellum, plays a significant role in producing a discriminated response to the two spatial changes.

## 2 MATERIALS AND METHODS

### 2.1 Subjects

Approval for this study was obtained in advance from the Ethics committee of the Ruhr University Bochum (Registration no.: 17‐6183). All participants gave written consent prior to participating. For the fMRI study, 27 (12 male) healthy adult subjects were recruited from the local student population, with a mean age of 24.3 years. For the EEG‐study a total of 28 healthy right‐handed subjects (11 males) were recruited from the local student population. This cohort had a mean age of 22. All subjects were self‐reported non‐smokers and medication‐free.

### 2.2 Stimuli

**Figure 1:**
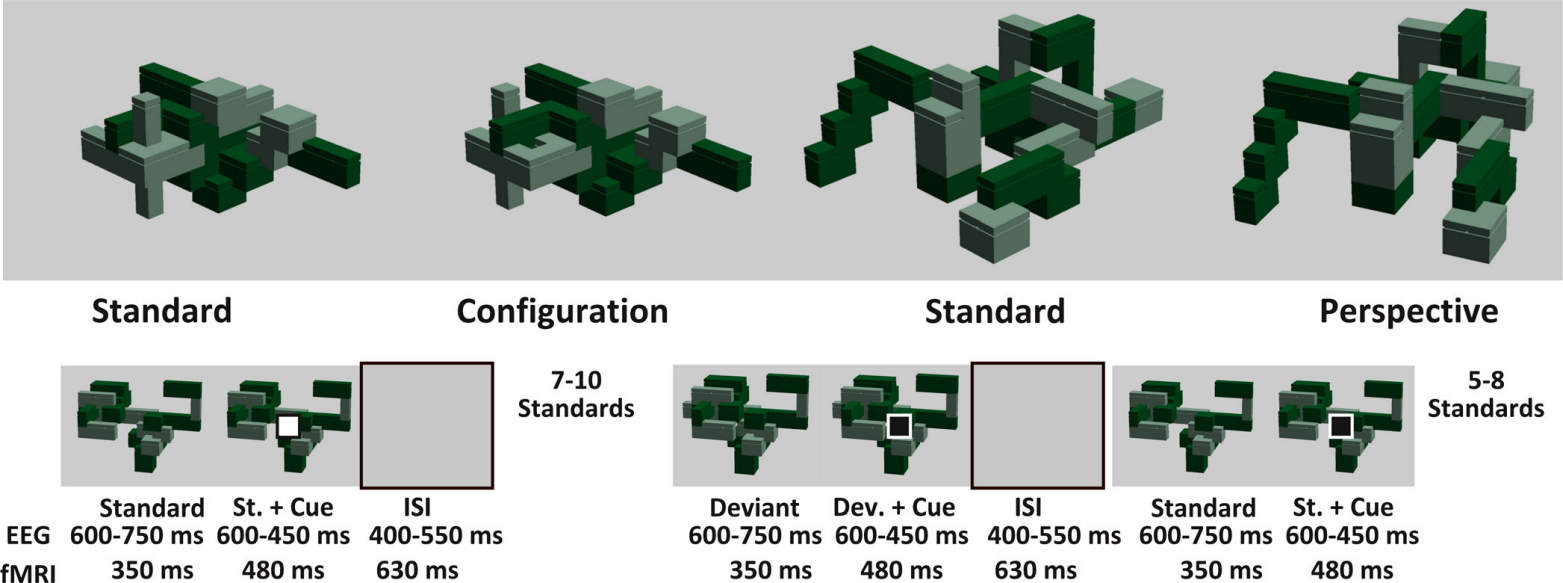
Examples of stimuli and structure of the oddball‐paradigm*. Top*: Standard images with their corresponding changes (deviants) in configuration or perspective. *Bottom*: Timing of the image presentation. Standard images were shown repeatedly, each time with a distracter cue (a small square appearing in the middle of the screen after a brief interval), to which the subjects were to respond with one of two buttons, depending on the color of the square. After 7–10 repetitions of the standard images, the deviant images were shown, that contained either configurational or perspective changes. The timing and onset of the distractor cue did not change for these changed images. After the presentation of the deviant, the standard was again shown 5–8 times. After this, a new standard and its corresponding randomly occurring deviant were shown in the same manner, without a change in timing

The stimuli, consisting of 70 and 80 abstract, three‐dimensional structures for the EEG‐ and fMRI‐study, respectively, were created using LEGO™ Digital Designer (The LEGO group, Billund, Denmark), each with one of two deviant versions (see Figure 1). Each structure was composed of 30–60 bricks in two shades of green. Top surfaces were covered using flat bricks, to avoid the structures being recognized as LEGO™‐components. In the context of the oddball paradigm, these structures served as standards, each of which was assigned one of two types of spatial change, which were implemented in the corresponding “deviant” versions of the standard. The configurational deviant was created by selecting a subset of the structure and either rotating it by 90° or 180°, or moving it by 4–12 possible adhesion points that are characteristic of LEGO‐bricks. The perspective deviant was created by centering the camera on a brick at the center of the structure and rotating the view‐angle by approximately 10° toward the left or right. The position in which configurational changes took place (in the left or right visual hemifield), as well as the view‐rotation (left or right), was balanced. The structures were presented in four blocks (with each block containing 20 structures on average). For the fMRI‐study, the images were presented at a rate of 1.66 s, each remaining visible for 830 ms, with an equally long interstimulus interval.

To ensure the subjects were looking at the structures, a distractor task was implemented. This consisted of a small black or white square (which we refer to as the cue) that appeared shortly after (on top of) the images. The task was to press one of two buttons, depending on which color this cue had. For the fMRI‐paradigm, this cue appeared after 350 ms. For the EEG‐study, the cue appeared later to avoid contaminating the event‐related potentials (ERPs) with motor responses, appearing between 600 and 750 ms after the image, and staying on screen together with the image of the structure for 450–600 ms. The interstimulus interval lasted for 400–450 ms. The cue color was pseudorandomized, to ensure that both black and white cues were presented equally often for all conditions.

Each standard was presented between 13 and 18 times, with the deviant occurring after the seventh and before the 12th presentation. After the 13–18 presentations of the standard, a completely new standard appeared without any interruption (unless there was a break). This number of presentations before a new standard was shown, as well as the position of the deviant was also pseudorandomized, and was the same for every participant, as was the order in which the structures were presented. For the fMRI‐study, all stimuli were presented using MR‐compatible LCD‐goggles, with an 800 × 600 pixel resolution, whereas for the EEG‐cohort a 40 cm monitor and a 1,024 × 960 resolution were used. The experiment was programmed using the Presentation software (Neurobehavioral Systems, Berkeley, CA).

### 2.3 Procedure

For the fMRI‐study, participants were instructed to remain as still as possible throughout the experiment and were given a verbal explanation of the task they were to complete while in the scanner. This instruction mentioned that they would be seeing a number of images, after which a small square would appear in the middle of the screen, to which they were to respond with one of two buttons using their right index and middle fingers, respectively. They were also informed that prior to the task, an anatomical scan would be done. After being placed on the scanner‐table, and while already wearing noise‐canceling headphones and earplugs, their head was further stabilized by padding out any remaining space. Before the start of the experiment, they were given a short training example of the task.

For the ERP‐study, participants were fitted with a 64‐Ag/AgCl‐electrode cap (Brain Products GmbH, Munich, Germany), which was secured with a chin‐strap and configured according to the 10% extension of the international 10–20 system. All electrodes were referenced to FCz, with the ground located at AFz. The electrodes were filled with electrode‐gel, and impedance was kept below 5 kΩ. After that, the participants were shown how their muscle activity would disturb the EEG‐signal and asked to be as relaxed as possible throughout the experiment. Subsequently, the eye‐tracker of the model “Eyelink 1000” (SR Research, Ottawa Canada) was calibrated and validated. The instructions for participants in the ERP study were the same as those for the fMRI study, and similarly, before the start of the experiment, training was provided.

After the experiment, the subjects were given a short questionnaire regarding the extent to which they noticed the spatial changes. These questions asked whether they noticed either of the deviant types, whether they focused on them, tried to identify them, or waited for the changes. Furthermore, if the participants noticed any of the changes, they were asked whether they did so already in the first block, to gain a rough indication of how alert participants were with regard to the passively perceived changes.

### 2.4 fMRI protocol

Data were acquired using a Phillips 3.0 Tesla Achieva X scanner and a 32‐channel head coil. First, the anatomical images were acquired using an MPRAGE‐sequence (TR = 8.5 ms, TE = 3.9 ms, voxel size = 1 mm^3^ isotropic). The functional volumes were acquired in four blocks of gradient‐EPIs (TR = 3.2 s, TE = 35 ms, flip angle = 90°, FOV = 192 × 224 × 150 mm^3^, 50 slices, with a thickness of 3 mm, voxel size = 2 × 2 × 3 mm^3^, 160, 167, 167, and 169 volumes, in addition to five preceding dummy‐scans, for the four blocks, respectively).

### 2.5 Image preprocessing and univariate analysis

Preprocessing, as well as the univariate analyses, were performed in SPM12 (Friston, Ashburner, Kiebel, Nichols, & Penny, 2007). The functional images were first subjected to slice timing correction, referenced to the 25th slice, after which the images were unwarped and motion corrected. The images were co‐registered and normalized to the SPM EPI‐template, after which they had a voxel size of 2 × 2 × 2 mm^3^, before being smoothed with a full‐width at half‐maximum of 6 mm kernel. First‐level model estimation was done per scanning block, with the first presentation of a completely novel standard, as well as the two deviant types containing the perspective and configurational changes forming their own regressors. All other image presentations, which were simply repetitions of the standard, formed one single regressor. Furthermore, the six motion parameters (three for rotation and three for translation) were added as nuisance regressors. We omitted the color of the cue that was used, as this was counterbalanced such that an equal number of black/white cues were presented across conditions in each block. For each subject, the main contrast of interest, namely the difference between perspective and configurational changes was calculated across the whole brain. Secondary contrasts (the results of which can be found in the supplementary materials) also compared the deviants against completely novel structures, as well as repeated items.

The second level model was run on these contrast images, where age, gender, and the results of the post‐experimental questionnaire regarding the extent to which subjects focused on the images were added as covariates of no interest. Significance thresholds for cluster formation were set to *p* < .001 uncorrected, with a minimum cluster size of *k* > 10, and final significance was based on the cluster‐size corrected for family‐wise error (FWE), at *p* < .05. In the cases where we employed small volume correction for hypothesis‐driven regions of interest, these were based on maximum tissue probability atlas from the OASIS project, included in SPM12 (and labeled by Neuromorphometrics Inc., Somerville, MA, under academic subscription).

### 2.6 Multivariate analysis

Single‐trial estimation of the configuration and perspective changes were done employing the least‐squares separate method (LSS; Mumford, Turner, Ashby, & Poldrack, 2012; Turner, Mumford, Poldrack, & Ashby, 2012), where a separate model is estimated for each individual trial, with the to be estimated trial forming its own regressor. We used the most recent proposition, the LSS‐N approach (Abdulrahman & Henson, 2016), where all trials other than the target‐trial are modeled in the same way as was done in the univariate model. Thus, new standards, repeated standards, and the two deviant types formed their own regressors, and motion parameters were included as before, in addition to the single‐trial regressor. Because we wanted to minimize any differences between the deviants that might have come from (non‐spatial) differences between their corresponding standards, we further modeled and subtracted from these deviant estimates the seventh presentations of each standard (the last before a deviant could possibly occur).

After having found activation in the right hippocampus in the univariate analyses, we used multivariate analyses to further explore the hippocampal involvement in passive spatial change perception. Briefly, we used a correlational classifier to identify subject‐specific hippocampal clusters that were most successful at differentiating between the two deviant types, followed by an informational connectivity analysis (Coutanche & Thompson‐Schill, 2013), using these clusters as seeds. Note that we intentionally overfitted the classifier to identify a seed‐region with high informational content, since the goal was to test whether the decision values would correlate with those of other regions, rather than simply demonstrating informational content in the hippocampus. A further point we wish to make at this stage is the fact that the correlational classifier that is employed in informational connectivity may not necessarily be sensitive to the univariate differences in activation (Walther et al., 2016).

We sought to identify which area in the right hippocampus was most successful at differentiating the two deviants by employing a searchlight approach (Kriegeskorte, Goebel, & Bandettini, 2006). For this, a sphere with a 4‐voxel‐radius was placed around each voxel in the right hippocampus of the unsmoothed, normalized beta‐maps, and all voxels contained therein were subjected to a correlational classifier, described as follows: The voxel pattern of each individual trial (e.g., a configurational deviant presented in block 1), was first correlated with its corresponding “prototype,” consisting of the mean pattern of all trials sharing the same condition label, but which were derived from other scanning blocks (e.g., the mean pattern of configurational deviants obtained over blocks 2, 3, and 4). This correlation value was then compared against the correlation with the prototype of the opposite condition (e.g., the mean pattern of perspective deviants derived from blocks 2, 3, and 4). The difference between the correlation with the prototype of a trial's true label (e.g., configurational deviants), and that of the other prototype (e.g., perspective deviants) will be positive if a trial can be correctly classified based on its pattern, and negative if classification is incorrect. Repeating this procedure for all trials, the resulting ratio of correct/incorrect classifications minus chance‐level was saved to the central voxel of the searchlight sphere. After obtaining these values for all voxels contained in the hippocampal mask, the resultant accuracy values were smoothed using a 6 × 6 × 6 mm^3^ Gaussian kernel, to be able to identify the most coherent area of high informational content within the hippocampus, rather than several fragmented ones. For each subject, the 50 voxels with the highest classification accuracy were selected to form a subject‐specific mask. For one subject, this procedure did not yield a cluster capable of classifying the two deviant types above chance, which was therefore excluded from the subsequent analysis.

We then wanted to test which networks or regions might be linked to these hippocampal seed regions. For this, we employed a second searchlight analysis coupled with the informational connectivity approach outlined by Coutanche and Thompson‐Schill (2013). This also involves the correlational classifier described above. However, in this instance, the trial‐by‐trial decision values are extracted from a seed‐region (here the subject‐specific hippocampal cluster), which are then correlated against the decision values of searchlight spheres (with a 4 mm radius) covering the rest of the brain, using Spearman's rank correlation. The resulting correlation values between the seed regions and searchlights decision values are then saved to the central voxel of the searchlight sphere. These correlations were then evaluated using a 1,000‐fold permutation test on a mask of superior parietal cortices, cerebellum, and left medial temporal lobe in the way described by Coutanche and Thompson‐Schill (2013), using as significance threshold the non‐parametric *p*‐value of *p* < .05. These analyses were performed using customized scripts from both the decoding toolbox (Hebart, Görgen, Haynes, & Dubois, 2015) and the RSA‐toolbox (Nili et al., 2014).

### 2.7 Preprocessing and analysis of ERP and eye‐tracking data

The raw data and event‐times were exported to Matlab, where they were then filtered using a bandpass filter of 0.5–70 Hz (high‐ and low‐pass, respectively), after which the data were referenced to the common average and segmented into 1,200 ms long epochs (including a 200 ms baseline). Besides the trials in which a configurational or perspective change was implemented, we also extracted all epochs belonging to first presentations (meaning completely new items), and second presentations (repetition condition) of each of the standards, which were used in secondary contrasts found in the supplementary materials. At this point, every trial was baselined to the 200 ms window before stimulus onset. Trials were then flagged as likely artifacts if they displayed maximum voltage steps of 50 μV, or an epoch‐wide maximal and minimal amplitude difference of 150 and 0.5 μV. After visual inspection of these trials, those deemed as artifacts were removed. After this, independent component analysis, using the fast ICA algorithm in EEGLAB, was performed to decompose the signal. Components displaying the typical pattern elicited by eye‐blinks were then weighted to 0 in the signal. Finally, the data were baselined to the 200 ms again.

An exploratory comparison between the deviants, new standards, and repeated standards was done on the timespan of 50–600 ms the results of which can be found in the supplementary materials. However, the main contrast of interest was the comparison between configurational and perspective changes. This was focused on the P300, due to its role in updating particularly in passive visual perception (Polich, 2007), and possible dependence on hippocampal function (Knight, 1996). This analysis was done using permutation tests with 5,000 iterations as implemented in the mass univariate ERP toolbox (Groppe, Urbach, & Kutas, 2011). The average amplitudes of all electrodes in three time‐windows covering the P300 component were used for this analysis. These spanned 300–400, 400–500, and 500–600 ms after the corresponding presentation of the deviants. The permutation test was done using an alpha level of 0.01.

Given the significant differences found here between the deviants, these time‐windows were then also subjected to a comparative source analysis using linearly constrained minimum variance on the basis of a 15 × 18 × 15 source‐grid spaced 1 cm apart and the boundary element head model provided as part of the fieldtrip toolbox (Oostenveld, Fries, Maris, & Schoffelen, 2011). The spatial filter was constructed based on all trials belonging to either of the deviant conditions and separately for each of the three time‐windows analyzed. The source estimates created on the basis of the two deviants were then contrasted, and a threshold of *p* < .05 was used to give an indication as to which regions were most likely to generate the significant differences at the electrode level.

The eye‐tracking coordinate data were imported into EEGLAB using the Eyetracking plug‐in (Dimigen et al. 2011), where they were epoched and averaged within the respective conditions. The saccades were detected automatically in the time‐series of gaze‐coordinates using a visual angle/pixel of 0.032, calculated on the basis of the subject being seated 70 cm from the screen, which had a width of 40 cm and a 1,024‐pixel resolution. For coordinate changes to be counted as a saccade, they were required to have a minimum duration of 8 ms. Subsequently, a moving average was used to count the number of saccades found at each time‐point within a 100 ms time‐window. This time series was then tested for significant differences between the two spatial deviants, as well as novel standards, and repeated standards using both maximum‐statistic, as well as cluster‐based permutation tests from 0 to 1,000 ms relative to stimulus presentation. The cluster formation threshold in the cluster test was set to *p* < .05.

## 3 RESULTS

### 3.1 Superior parietal cortex, hippocampus, and cerebellum respond differentially to observer‐dependent and allocentric change

**Figure 2:**
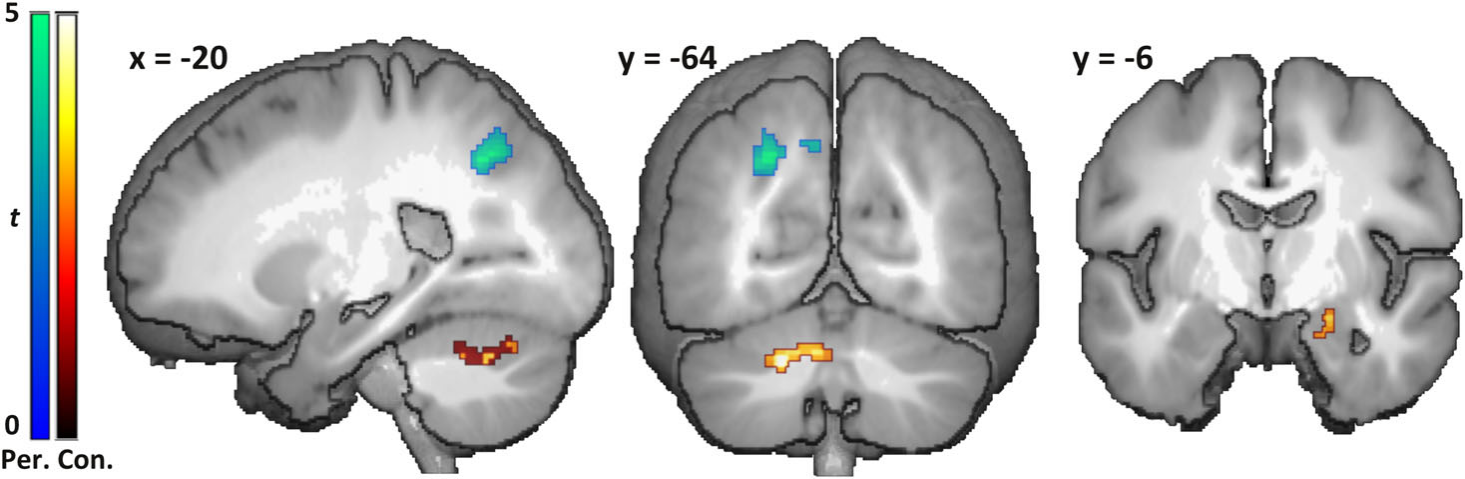
Univariate results for main contrast show distinct responses for configurational and perspective changes. Clusters of activity estimates found to be significantly different between configurational (Con., warm colors) and perspective changes (Per., cool colors), rendered onto the mean anatomical image. *x* and *y* coordinates refer to MNI‐coordinate of sagittal and coronal planes, respectively

**Figure 3:**
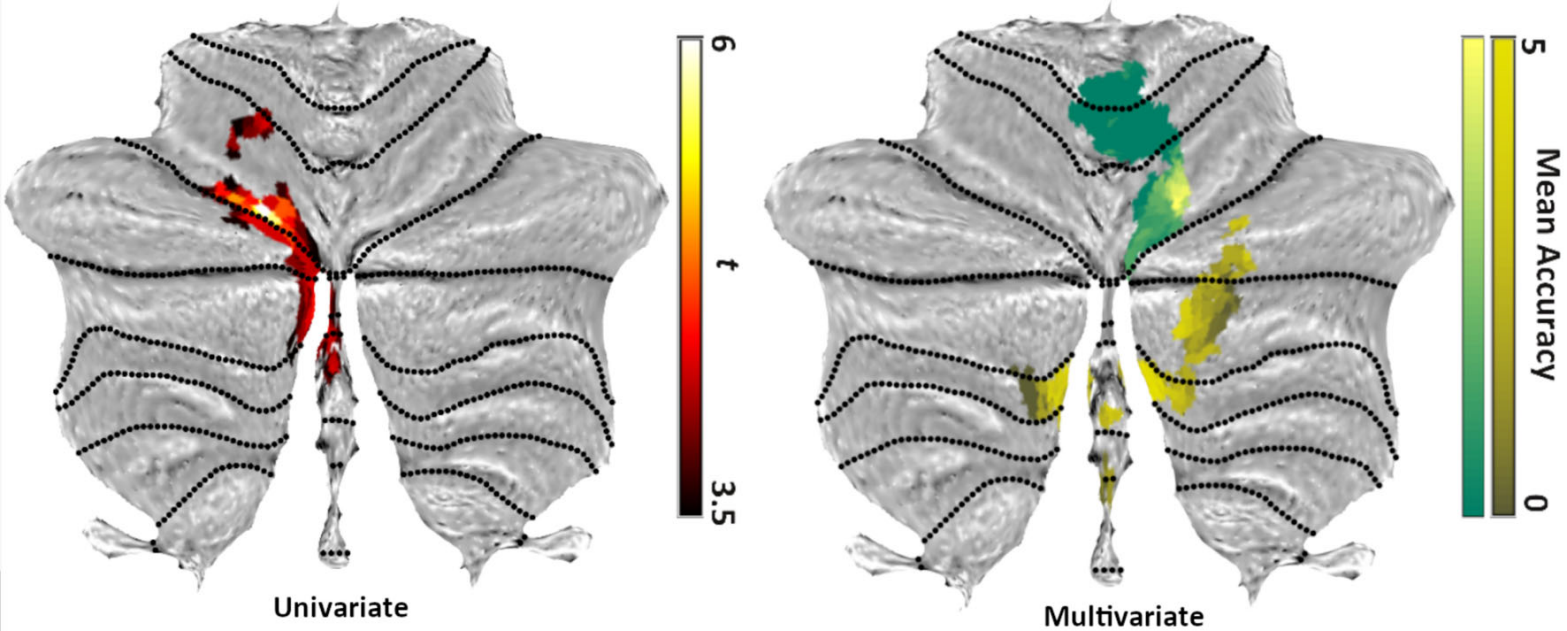
Cerebellar activations in univariate analyses and cerebellar classification accuracies in multivariate analyses. *Left*: Cluster of differential cerebellar activity in *Configuration* > *Perspective* contrast, rendered onto the flatmap of the SUIT atlas (Diedrichsen, 2006; Diedrichsen, Balsters, Flavell, Cussans, & Ramnani, 2009; Diedrichsen & Zotow, 2015). *Right*: The average accuracy, minus chance, of classifying perspective and configurational deviants, obtained using an 8 mm searchlight across the two clusters that were found to be significantly correlated with the hippocampus (yellow and green areas correspond to the yellow and green clusters in Figure 4)

In the fMRI‐data, the main contrast of interest was that between activity elicited by unexpected changes in viewpoint, and by changes in configuration. For this, a whole‐brain analysis was conducted that yielded three significant clusters (Figure 2; Table 1). The largest of these was in the cerebellum, where a cluster extending across left lobule VI, Crus 1 and 2, and lobule VIII on the vermis, was significantly more active for configurations compared to the view‐point changes (Figures 2 and 3 left flatmap). The same held true for a small cluster in the right anterior hippocampus, which survived FWE‐corrected thresholds after applying the hypothesis‐driven small volume correction for the right hippocampus (Figure 2). By contrast, a larger cluster significantly more active for viewpoint changes was found in the left superior parietal lobule, extending into the left precuneus.

**Table 1:** Univariate results for the contrasts between deviants and new conditions

				MNI coordinates		
Regions	Hemisphere	Cluster size	*Z*_max_	*x*	*y*	*z*
*Config. deviant > Persp. deviant*						
Cerebellum sixth & eighth lobule, crus I	L/Vermis	202	4.6	−14	−66	−28
Hippocampus	R	11	+4.53	18	−10	−20
*Config. deviant < Persp. deviant*						
Superior parietal lobule/precuneus	L	181	4.44	−8	−68	44

+ denotes significance at peak level with FWE‐corrected *p* < .05, after small volume correction for the right hippocampus.

Evaluating the post‐experimental questionnaire, which addressed to what extent subjects noticed the small changes to the repeatedly shown structures, indicated that most (24 of the 28) subjects noticed the configurational changes at some point. Just under half (13) of the cohort reported having been aware of the perspective changes, and a total of 17 subjects reported having been aware of any of these changes (perspective and/or configuration) already early on, in the first block. A total of 15 subjects reported having concentrated on, waited for, or tried to identify the change. To test to what extent brain activity was modulated by these variables, we summed the positive answers in this questionnaire and used it as a covariate in the above contrast. However, this variable did not have a significant impact on activity in any brain regions, and also a statistical threshold uncorrected for multiple comparisons did not yield activity estimates comparable to those reported for the main effect. The only regions found to be significant at this threshold were the right superior frontal gyrus, and the left medial frontal cortex, where activity was higher for viewpoint changes in subjects that were more aware of the spatial alterations.

Next, we tested whether the parietal areas that we found to be significantly more active for view‐changes than configurations might be explained by a repetition‐dependent increase in activity (Brodt et al., 2016; Brodt et al., 2018; Gilmore et al., 2015; Schott et al., 2019). For this, we reestimated the first‐level model including a parametric modulator containing the relative position in the repetition sequence of a given standard. The parietal activity remained significant despite accounting for the repetition effects.

### 3.2 Hippocampal and cerebellar activity are linked on a trial‐by‐trial level

Next, we wanted to investigate whether the activity between these different regions was linked, particularly how the hippocampal responses were related to other brain regions. For this, the informational connectivity analysis (Coutanche & Thompson‐Schill, 2013) outlined previously was run on single‐trial estimates of the neural responses to the structures containing either a change in configuration or viewpoint.

**Figure 4:**
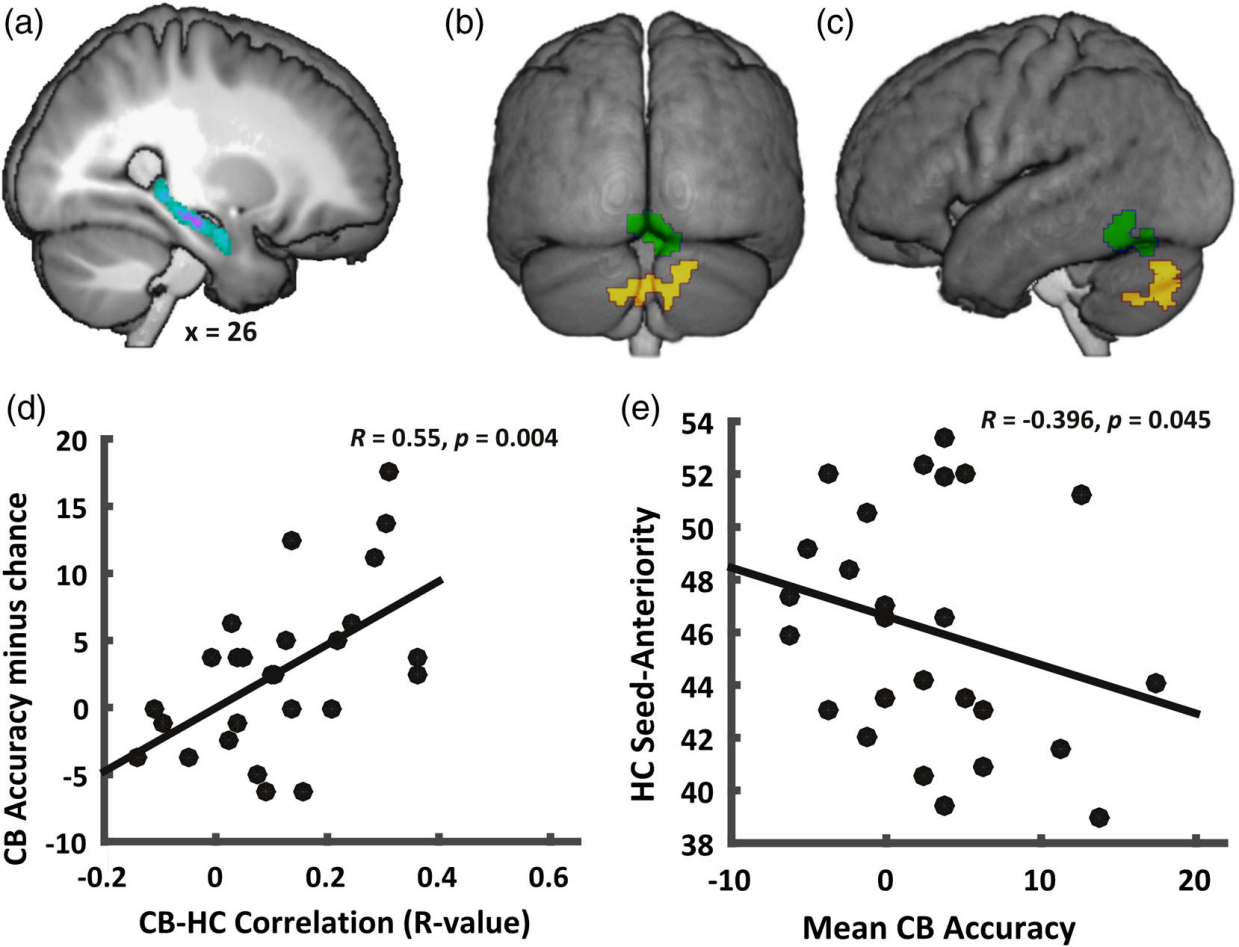
Multivariate results demonstrate a cerebello‐hippocampal connection at an informational level*. Top*: Map of hippocampal seed regions summed across participants (a); the two clusters (green: vermal lobule IV–VI; yellow: lobule VIIb and Crus 1 and 2) informationally connected to seed regions shown from posterior view (b), as well as the left sagittal view (c). *Bottom*: Cerebellar VIIb classification accuracy versus cerebello‐hippocampal connectivity estimates (d), and correlation between the location of peak classification accuracy in the hippocampus (anterior–posterior axis) and mean searchlight accuracy in cerebellar VIIb cluster across participants (e)

At the group level, two clusters emerged as significantly connected to the hippocampal seed‐classifiers, both of which were in the cerebellum (see the right flatmap in Figure 3 and Figure 4b,c). The first cluster was located vermally in lobule IV, V, and VI, and the second in lobule VIIb bilaterally, as well as the right Crus 1 and 2, and to a lesser extent in lobule VIII, bilaterally, and on the vermis (see Figure 3 right flatmap and Figure 4b,c).

While the first of these (the cluster spanning lobules IV–VI, shown in green in Figures 3 and 4) did not show a significant above chance classification rate (*t*[25] = −1.22, *p* = .23), the cluster in lobule VIIb did (*t*[25] = 2.25, *p* = .03). We then aimed to rule out that this connectivity effect was a result of low classification accuracy, which would be the case if for example individual trial‐estimates are affected by global noise, making them stand out, and be more likely to be misclassified in both, seed‐ and searchlight‐classifiers. This could not only be ruled out but in fact, the opposite was the case: subjects where a cerebellar classifier performed better, also displayed a higher degree of connectivity with the hippocampal seed‐region (*R* = 0.55, *p* = .004, see Figure 4d).

Since in these analyses, the seed region consisted of a subject‐specific classification maximum within the hippocampus, we next tested whether the location along the hippocampal anterior–posterior axis followed a systematic pattern. We found that the average voxel‐location of these seed‐clusters in the hippocampus were correlated with the average accuracies of the searchlight classifiers contained in the cerebellar cluster (*R* = −0.3955, *p* = .045, see Figure 4e), such that a higher mean cerebellar classification accuracy was linked with a more posterior hippocampal classification maximum for differentiating neural responses to viewpoint and configurational changes.

### 3.3 Cerebellar pattern classification between spatial changes is not related to motor performance in distractor task

One alternative and feasible explanation for this connectivity was that subjects might have changed their motor behavior in the distractor task during the occurrence of the spatial changes, even though they were not relevant for the task. For this, we analyzed the behavioral data of the distractor task, where subjects were required to respond to a small square appearing after every image, depending on its color. Due to a malfunction in the recording set‐up, responses for the first nine subjects were not available. The other subjects performed with an accuracy of 99% (*SD*: 0.2), across the 1,236 trials in the experiment.

Configurational and viewpoint changes yielded very similar response latencies of 0.39 (*SD*: 0.031), and 0.399 (0.037) s, respectively. For reference, the response latency for standards that had been shown for the seventh consecutive time was 0.396 (0.034) s, whereas the presentation of completely new structures (new standard images) yielded an average response latency of 0.431 (0.04), which was significantly longer than the above (*F*[3,51] = 24.863, *p* < .001). Importantly, the two spatial change conditions did not differ from each other in a significant way.

Nonetheless, we tested whether, on a trial‐by‐trial basis, an increased response‐latency might relate to the performance of the classifier. For this, we *z*‐transformed latencies within participants and replaced non‐response‐trials with the maximum response‐latency of a given subject. All trials from all participants were then collectively tested for a correlation between the standardized latency and raw decision values (i.e., which condition the classifier assigned to a given trial, and with which certainty), absolute decision values (i.e., only the certainty, regardless of accuracy), as well as accuracy values (i.e., how correct the classifier was for every trial). In neither cerebellar cluster, nor the hippocampal seed, were any of these correlations significant.

In conclusion, the fMRI data demonstrated that the anterior hippocampus responded differentially to the passive perception of feature‐relationships that resulted from a change in viewpoint or configuration, in that its BOLD‐response was enhanced for the latter. The same held true for a broad cluster in the cerebellum, whereas the superior parietal cortex responded significantly to view‐point changes, in line with our hypothesis.

At the multivariate level, no single region in the hippocampus was found that carried informational content in a way that was significant across subjects. However, the approximated single‐subject classification maxima in the right hippocampus were correlated to cerebellar regions in a way that also related to their location along the anterior–posterior hippocampal axis.

### 3.4 Scalp‐topographical amplitudes during the P300 differentiate between spatial changes and localize to parietal and temporal areas

Looking at the ERP‐data obtained from a different cohort, but using a very comparable paradigm, we focused on the P300 component, thought to reflect updating processes (Jeon & Polich, 2001; Polich, 2007; Polich & Squire, 1993) potentially related to hippocampal function (Brankačk, Seidenbecher, & Müller‐Gärtner, 1996; Knight, 1996). We also conducted an epoch‐wide analysis of contrasts with repeated items that are available in the supplementary materials.

**Figure 5:**
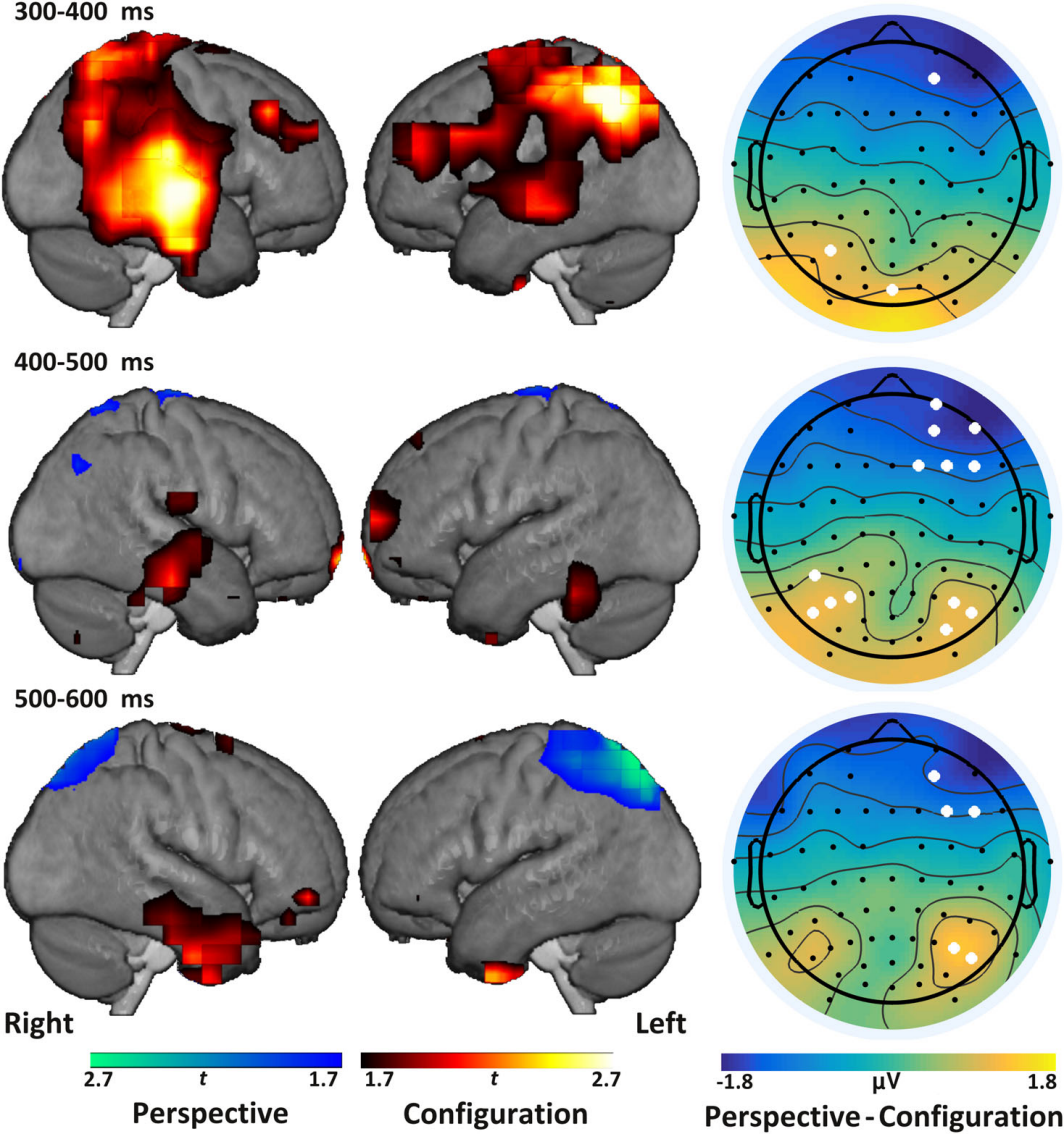
Source estimates of differences in P300 between deviants partially match results from fMRI study. *Left and center column*: Interpolated source estimates for the two deviant types in three average time‐windows of the P300, rendered onto the mean normalized anatomical images obtained in the MRI study. Clusters are obtained by thresholding *t* values of contrasted source estimates at *p* < .05 (uncorrected) for configuration versus perspective deviants. *Right column*: Topographies of different amplitudes of perspective–configuration deviants. White dots indicate electrodes significant at *p* < .01 in permutation tests for maximal *t* values

Using permutation tests, three 100 ms time‐windows spanning this component were tested for clusters of electrodes displaying a significant differential mean‐amplitude between the two spatial changes. Significant differences between the two deviants at *p* < .01 were found in all three time‐windows (Figure 5). These consisted of right frontal electrodes and a progression from left to right parietal electrodes across the three segments. The corresponding source‐estimates of the first segment (300–400 ms, top row in Figure 5) displayed increases in source power in parietal and right temporal areas for configurational, relative to perspective changes. Subsequently, in the 400–500 ms time‐window the parietal contribution was almost reversed, in that viewpoint changes now showed very small clusters of relative increases (middle row in Figure 5). Stronger estimates for the configurational changes were found in a distributed network of frontal, temporal, and cerebellar and/or inferior occipital areas. The last time‐window (500–600 ms, bottom row in Figure 5) appeared to corroborate the findings from the univariate fMRI data the most, in that perspective deviants were found to be linked with an increase in parietal generation, and a now more pronounced contribution of anterior temporal areas to configurational changes.

Together, the source analyses indicate that configurational novelty elicited a progression from parietal to temporal cortices. Within the temporal areas, the most likely sources appeared to progress from more posterior to anterior areas over the second and third time‐windows. By contrast, perspective changes were most strongly linked with an increased parietal source estimate in the final time‐window.

### 3.5 Saccade‐numbers do not differ between spatial changes

An important point to consider in the fMRI‐results described above is the possibility that differential activity in superior parietal cortex, cerebellum, or hippocampus might potentially also have arisen from differences in eye‐movements. While we did not collect eye‐movement data for the fMRI cohort, these data were available for the ERP‐cohort. With this in mind, we compared the moving 100 ms average of saccade numbers between the different types of visuospatial novelty (configurational changes, viewpoint changes, and entirely novel structures), and a non‐novel baseline (for which we chose the second presentation of an image). The resulting time‐series was then tested for significance between the deviants, which was the main contrast of interest. For reference, the saccade numbers for completely new standards, as well as repeated standards were also compared to each other and the deviants. These contrasts were tested for significance using two types of permutation tests taking into account cluster size or maximum statistics.

**Figure 6:**
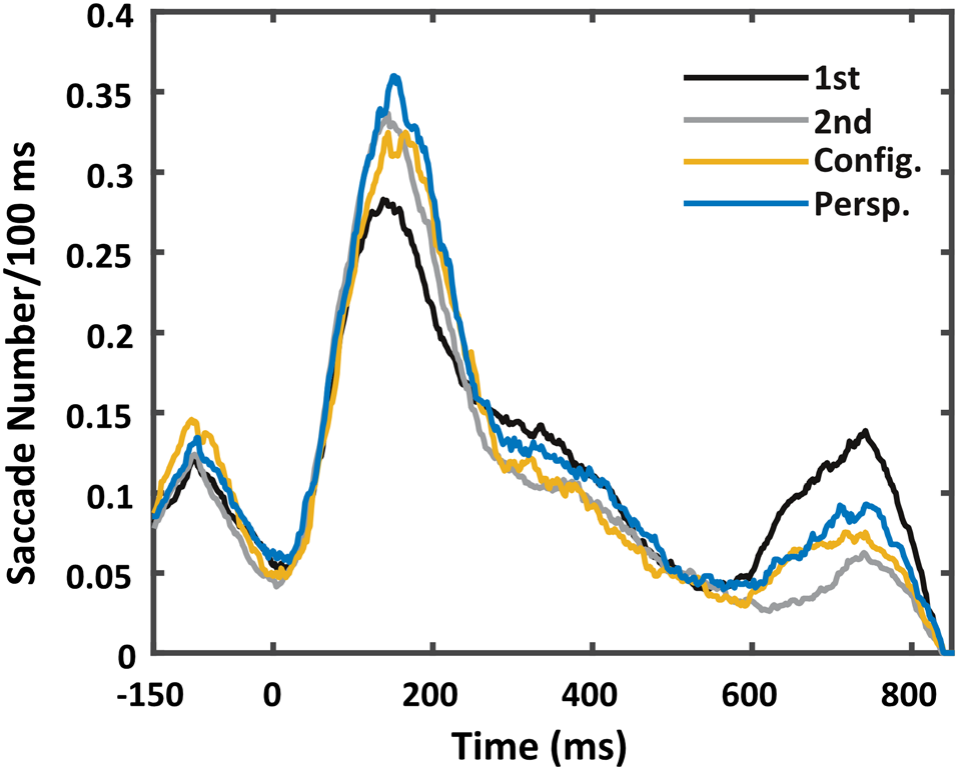
Saccade numbers are not significantly different between deviants. Moving average saccade numbers across 100 ms for new and repeated standards (“1st” and “2nd” presentations, respectively), as well as the configurational and perspective changes (“Config.” and “Persp.”) over time, where 0 represents the onset of the respective image presentation

Between new items and the baseline, a brief significant difference was observed from 650 to 790 ms (Figure 6), such that for new items subjects made more saccades than for repeated items. This was similar in perspective but not configurational deviants. Despite the fact that perspective deviants elicited an increase in saccades relative to the baseline at this late time‐point, there were no significant differences in saccade numbers between the two deviants.

## 4 DISCUSSION

In the two studies presented here, we investigated the neural response, measured using fMRI and EEG, to passively perceived visuospatial novelty that arose from either observer‐dependent (egocentric) or allocentric, configurational changes of abstract structures in an oddball‐like paradigm. On the basis of previous studies indicating that the perception of novel high‐resolution bindings (Aly et al., 2014; Yonelinas, 2013) as well as identifying changes across different viewpoints (Barense et al., 2010; Lee et al., 2012; Lee, Buckley, et al., 2005) relies on the hippocampus, we focused on this region in particular. In the fMRI‐data, univariate analyses indicated that perspective changes elicited stronger activity in the left superior parietal cortex, compared to configurational novelty. By contrast, configurational changes elicited activity in a small cluster in the anterior hippocampus, more so than perspective changes. Source analyses of the ERP‐data confirmed that these areas are involved in the processing of these types of visuospatial novelty, indicating in turn that anterior temporal and parietal responses to configurational and perspective changes, respectively, support the generation of P300 amplitudes.

A somewhat surprising finding in the fMRI data was that the cerebellum responds to configurational changes when compared to perspective changes. When scrutinized on a multivariate level, the data suggest an involvement of a cerebello‐hippocampal link in differentiating spatial changes, depending on the reference frame in which they happen. This connection furthermore was related to the location of maximal classification accuracy on the anterior–posterior axis of the right hippocampus: We found a significant tendency for higher cerebellar classification‐accuracy to be linked to a more posteriorly located hippocampal classification maximum.

### 4.1 The hippocampus in passive perception

Overall, our results add further evidence to recent suggestions that the hippocampus is involved in perception, as suggested by others (Lee et al., 2012), specifically, that the hippocampus responds to high‐resolution changes (Aly et al., 2013). In the present study, this was the case when these changes consisted of a spatial reconfiguration, but not when the changes arose from a perspective change. However, given previous reports on the subject (e.g., Gomez, Cerles, Rousset, Rémy, & Baciu, 2014), it appears unlikely that the hippocampus did not react at all to perspective changes. Rather, we believe that the configurational changes elicited a more profound updating process in the hippocampus compared to the viewpoint changes that arguably are more subtle, and even have little behavioral consequence (Hollingworth & Henderson, 2004). On the basis of pattern‐separation and completion accounts of hippocampal function, it seems plausible that the former process would be more involved in the configurational changes and the latter in viewpoint changes. This would mean that configurational changes, despite being less salient on a perceptual level than perspective changes, can drive a detonator‐mechanism leading to a stronger hippocampal response (Bakker, Kirwan, Miller, & Stark, 2008; Lacy, Yassa, Stark, Muftuler, & Stark, 2011; McNaughton & Morris, 1987; Yassa & Stark, 2011) and possibly to pattern separation. Perspective changes, on the other hand, may be scaled with the hippocampal response in a more linear manner, due to the fact that their novelty is more likely to lead to a pattern completion response. This interpretation is in line with our observation that more subjects of the fMRI‐cohort reported having noticed the configurational changes, rather than the perspective changes.

### 4.2 A novel cerebellar‐hippocampal connection for visuospatial information processing

A particularly significant finding of the data presented here is that different clusters in the cerebellum showed a response pattern comparable to the hippocampus. While this result might be interpreted as the cerebellum playing the role of an error‐detection‐device (Shadmehr, Smith, & Krakauer, 2010), both deviant types were equally unexpected, in that they occurred randomly. While eye‐movements might play a role in the cerebellar activity we found here (Ron & Robinson, 1973), the eye‐tracking data from the ERP cohort indicate that the two deviants did not elicit significantly different numbers of saccades at any point throughout the stimulus presentation. Furthermore, a recent study has demonstrated that, at least with regard to verbal working memory load, increases in cerebellar activity, specifically in lobules VI and VIIb, are largely independent of eye‐movement (Peterburs, Cheng, & Desmond, 2016). Finally, we also did not find a link between response‐characteristics of the distractor task and classification in the cerebellar clusters, which also speaks against a motor‐related interpretation of our findings. Instead, recent systematic evaluations of cerebellar activity across a large battery of tasks (King, Hernandez‐castillo, Poldrack, Ivry, & Diedrichsen, 2019; Stoodley, Valera, & Schmahmann, 2012) produced a pattern comparable to ours when subjects performed mental rotation. Specifically, the activity across the left intercrural fissure that we see in the univariate results (Figure 3 left flatmap), and the medial portions in lobule VIIb of the more posterior cluster in the multivariate connectivity results (Figure 3 right flatmap) align well with the data produced by King and colleagues (2019).

This finding can be considered in the context of reports that the cerebellum plays a critical role in hippocampal spatial updating in navigation (Iglói et al., 2015; Rochefort et al., 2011; Rochefort, Lefort, & Rondi‐Reig, 2013). In a study by Rochefort et al. (2011), mutant mice with cerebellar dysfunction were found to display unstable hippocampal place fields when the environment was changed in their presence. Similarly, Iglói et al. (2015) found different cerebello‐hippocampal connectivity patterns in human subjects, depending on whether they employed navigation strategies based on ego‐ or allocentric reference‐frames. These studies suggest that the cerebellum plays a crucial role in updating allocentric, as well as egocentric representations at the hippocampal level during navigation. As we have pointed out in Section 1, the terms ego‐ and allo‐centric used here cannot be directly transferred to the similarly named concepts in navigation, as the paradigm did not necessarily involve the creation of a cognitive map or self‐motion. However, the stimuli can be related to these concepts at the level of which reference frame they challenged (Filimon, 2015). This, we believe, would provide a common denominator between navigation and the perceptual processes addressed in the present study, where cerebello‐hippocampal connectivity might serve to identify the reference frame to which perceived novel feature‐relationships can be attributed.

The link between the average classification of the searchlights in the cerebellar cluster and the hippocampal anterior–posterior axis location of classification maxima may be of special interest for future endeavors on this topic. The functional implication of this hippocampal axis has been the subject of much debate (Poppenk, Evensmoen, Moscovitch, & Nadel, 2013). However, one theory suggests that the more posterior portion of the hippocampus is relevant for the processing of detail, while the anterior portion is relevant for gist (Evensmoen et al., 2013). This provides a compelling interpretative framework for our results. Assuming the cerebellum performs information preprocessing for the hippocampus, in that it identifies the precise features of the perceived novelty that should be updated in hippocampal representations, the outcome of this computation will determine the extent to which representational detail can be achieved in the latter. Therefore, the more involved the cerebellum is in differentiating (and thus the better its patterns differentiate between the two spatial changes), the more detailed the resulting hippocampal representations will be. According to the gist/detail‐account, this would elicit a more posteriorly located locus of hippocampal pattern differentiability. The assumption of the cerebellum passing information to the hippocampus is of course highly speculative and not supported by our data. However on the basis of electrophysiological studies demonstrating cerebellar influence in the hippocampus of cats (Newman & Reza, 1979), and monkeys (Heath, Dempesy, Fontana, & Myers, 1978; Heath & Harper, 1974), and especially the aforementioned studies showing that impaired cerebellar function has an impact on hippocampal responses in spatial tasks (Rochefort et al., 2011), we find this possibility bears consideration.

Related to this, to what extent a connection between the cerebellum and hippocampus in this context is direct (Arrigo et al., 2014), or indirect (Yu & Krook‐magnuson, 2015) cannot be specifically inferred from our data. It is possible that the parietal cortex plays an important role, given that both ERP and fMRI data indicated that this region is significantly more active during perceiving changes that arise from novel viewpoints. This will be discussed next.

### 4.3 The parietal cortex in passive perception

As already pointed out in Section 1, the medial parietal cortex has recently been found to likely complement the hippocampus in the establishment of memory representations, as it is sensitive to repetitions but suppressed during initial encoding of novel information (Brodt et al., 2016; Brodt et al., 2018; Gilmore et al., 2015; Schott et al., 2019). We did not find that the activity in the parietal cortex could be explained by the number of repetitions preceding the deviants, suggesting that the activity we see here is not merely a reflection of this reinstatement‐dependent behavior. Thus, above and beyond the elevated activity for familiar information, the parietal cortex appears to respond to viewpoint changes. What could this activity reflect?

The parietal cortex has been suggested to process transformations from visual to action, or motor representations (Murphy, Leopold, Humphreys, Welchman, & Murphy, 2016), a function that is probably subserved by the computation of viewer and object reference frames, both of which are dimensions to which parietal neurons are sensitive to (Chafee et al., 2008). Speculatively, we would argue the parietal cortex activity seen here might reflect the precise spatial transformation required to explain the view‐point deviant. Given that, the parietal cortex is also one of the major output targets of the cerebellum (Clower, West, Lynch, & Strick, 2001), it would be a good candidate for mediating the cerebello‐hippocampal connection outlined in the previous section. Interesting at this point is the observation that the source estimates of the ERPs elicited by configuration deviants indicated a transition from parietal to temporal areas. From the perspective of the two visual streams (Ungerleider & Mishkin, 1982; Mishkin, Ungerleider, & Macko, 1983), this could be argued to constitute a switch from the dorsal to the ventral stream for deviants that did not contain a change that could be achieved by a motor sequence or action (such as moving a few steps to the right or left). Indeed, more recent propositions that link these pathways to the hippocampus (Kravitz, Saleem, Baker, & Mishkin, 2011), align with these interpretations. Though the spatial resolution of our fMRI‐scans was too coarse to be certain, the anterior activity seen for the hippocampus might include the (pre‐)subiculum, which is suggested to support holistic visual processing due to its interaction with the parieto‐medial stream (Dalton & Maguire, 2017). High‐resolution fMRI may elucidate the precise locus of activity of these effects in the future.

Given these findings in the ERP data, indicating a potential exchange of information between the temporal and parietal areas, and the previously reported evidence for a parietal connection to both, hippocampal and cerebellar areas, why was the parietal cortex not featured in the informational connectivity analyses? It may be the case that a cerebellar (and hippocampal) response to perspective changes happens in circuits to which fMRI is less sensitive (Diedrichsen, Verstynen, Schlerf, & Wiestler, 2010). Thus, no connection to the parietal cortex (where the response was significant) could be detected. However, future studies could investigate this potential triad in spatial perception using more suitable imaging modalities.

## 5 CONCLUSIONS

In conclusion, our study demonstrates that a relationship exists between the cerebellum and the hippocampus, that is comparable to what has recently been reported in navigation, and which extends to the domain of perception of visuospatial information. Specifically, the cerebellum supports the delineation between allocentric and observer‐dependent novelty, an effect that is congruent with numerous interpretations of the cerebellar role in navigation. The demonstration of such a relationship in basic, passive visuospatial perception shows that the computation of information between these two structures may constitute a very fundamental process that can be expected to be implicated in a much larger range of brain functions than previously thought.

## CONFLICT OF INTEREST

The authors declare no potential conflict of interest.

## AUTHOR CONTRIBUTIONS

D.M.‐V. initiated the concept and strategy of the study that was further developed together with M.F.A.H.; the fMRI paradigm was developed by M.F.A.H., S.H., T.S.‐W., and M.T.; fMRI experiments were conducted by M.F.A.H. together with S.H.; EEG experiments were conducted by M.F.A.H.; data were analyzed by M.F.A.H. and interpreted by M.F.A.H. and D.M.‐V.; the manuscript was written by M.F.A.H. and D.M.‐V.

## Supporting information


**Appendix S1**: Supplementary Materials

**Table S1** Univariate results of the contrasts of deviants and new items against repetitions.

**Figure S1** Univariate results rendered onto mean anatomical image show visual cortical activity in response to new items, cerebellar and hippocampal activity for configurational (config.) deviants, and parietal activity for perspective (persp.) deviants.

**Figure S2** Condition‐averaged ERPs across different electrode‐subsets. Conditions plotted are the for new and repeated standards (‘1st’ and ‘2nd’ presentations, respectively), as well as the configurational and perspective changes (‘Config.’ and ‘Persp.’). Time‐point 0 represents the onset of the respective image presentation.

**Figure S3** ERP‐results show topographical differences between effects arising mostly in late time‐windows. Baseline‐subtracted topographies of time windows in which significant differences were found for novel (new) items, configurational deviants, or perspective deviants against baseline. White dots indicate electrodes significant at *p* < .05.

## Data Availability

The data that support the findings of this study are available on request from the corresponding author. The data are not publicly available due to privacy and ethical restrictions.
