# Supplementary material for: Cerebellar‐hippocampal processing in passive perception of visuospatial change: An ego‐ and allocentric axis?
Source: Hum Brain Mapp. 2019 Nov 15;41(5):1153–66. doi: 10.1002/hbm.24865 (PMC7268078; doi:10.1002/hbm.24865)
Supplement: Supplementary file 1 — Appendix S1: Supplementary Materials [file HBM-41-1153-s001.docx]

**Supplementary Materials**

Secondary contrasts for univariate fMRI analyses

In addition to the main contrast between the two types of spatial change, we also investigated how each of these compare to familiar standards (the regressor for which contained all images that had been repetitions of an image), and how entirely novel standards (thus the first presentation) compare in their response.

Here we present the results of the contrast between activity in response to novel standard as well as each of the deviants against the activity estimates for familiar, repeated items. For new items, we detected wide-spread, bilateral activity in occipital cortices, extending dorsally to the right superior parietal cortex, and ventrally to the fusiform gyri up to the inferior temporal areas. For the configurational deviants, activity was mostly found in cerebellar regions, particularly in lobules 6 and 8 (bilaterally), as well as in the vermis. Furthermore, we found significant peak activation on the border between the right anterior hippocampus and parahippocampal area. Finally, for perspective deviants, we found significant activity in the left superior parietal lobe. These results are presented in figure S1 and table S1.

**(Table S1 to be placed around here)**

In summary, we observed that compared to familiar images the hippocampus and cerebellum respond to configurational changes within items, whereas the parietal cortex responded to perspective changes. This reflects the differences in activity that we had found to be significant in differentiating the two spatial deviants. New items elicited mostly visual cortex and parietal activity.

**(Figure S1 to be placed around here)**

Exploratory ERP analyses and secondary contrasts

Complementing the main focus of the P300 and the comparison between the two deviants, we compared the ERPs (see figure S2) evoked by the perspective and configurational changes against the second presentation of the standard, which here served as the non-novel baseline. This was done on the entire epoch from 50-600 ms using permutation tests based on the maximum test statistic, that were deemed significant at *p* < 0.05.

**(Figure S2 to be placed around here)**

According to these tests, a number of significant differences were found across the broader time-window. The first of these occurred in all of the conditions containing any kind of novelty around 100 ms, corresponding to the P1-component, though this appeared to be largely due to different latencies of that component, with the baseline P1 peaking a little earlier than the novelty conditions. At 160 ms, right parietal electrodes showed significant differences for new items and perspective deviants, and at 180 ms this extended to middle occipital electrodes only for new items. At 274 ms, the level of the P2, all conditions differed significantly from baseline in parietal and frontotemporal electrodes, with topographical differences (meaning the spatial distribution of significantly different amplitudes among electrodes) between the deviants becoming prominent only over the course of the P300, at 350 ms, 400 ms, and 580 ms (figure S3). Taken together, the analysis of ERP-amplitudes revealed that the earlier components display largely similar effects for all types of visual novelty (that is new items, perspective, or configurational changes), with differentiable effects appearing to arise over the course of the P300 component.

**(Figure S3 to be placed around here)**

Figure Legends

Figure S1. Univariate results rendered onto mean anatomical image show visual cortical activity in response to new items, cerebellar and hippocampal activity for configurational (config.) deviants, and parietal activity for perspective (persp.) deviants.

Figure S2. Condition-averaged ERPs across different electrode-subsets. Conditions plotted are the for new and repeated standards (‘1st' and ‘2nd' presentations, respectively), as well as the configurational and perspective changes (‘Config.’ and ‘Persp.’). Time-point 0 represents the onset of the respective image presentation.

Figure S3. ERP-results show topographical differences between effects arising mostly in late time-windows. Basline-subtracted topographies of timewindows in which significant differences were found for novel (new) items, configurational deviants, or perspective deviants against baseline. White dots indicate electrodes significant at p < 0.05.
