# Supplementary material for: Cerebellar‐hippocampal processing in passive perception of visuospatial change: An ego‐ and allocentric axis?
Source: Hum Brain Mapp. 2019 Nov 15;41(5):1153–66. doi: 10.1002/hbm.24865 (PMC7268078; doi:10.1002/hbm.24865)
Supplement: Supplementary file 2 — Table S1 Univariate results of the contrasts of deviants and new items against repetitions. [file HBM-41-1153-s002.docx]

**Table S1.** Univariate results of the contrasts of deviants and new items against repetitions.

| **Regions** | **Hemisphere** | **Cluster Size** | | ***Z*_max_** | **MNI-Coordinates** | | |
| --- | --- | --- | --- | --- | --- | --- | --- |
|  |  |  | |  | *x* | *y* | *z* |
| *New > Baseline* |  |  |  | |  |  |  |
| Middle & Inferior Occipital G. | R | 320 | 5.19 | | 40 | -84 | 10 |
| Fusiform G., Inferior Occipital G. | L | 1585 | 4.92 | | -36 | -72 | -18 |
| Angular G., Superior Occipital G. | L | 138 | 4.7 | | -26 | -82 | 24 |
| Middle Occipital G., SPL, Angular G. | R | 324 | 4.62 | | 32 | -78 | 32 |
| Fusiform G. | L | 86 | 4.1 | | -32 | -42 | -12 |
| Inferior Occipital G., Fusiform G. | R | 95 | 3.77 | | 44 | -76 | -2 |
| *Config. Deviant* *> Baseline* |  |  |  | |  |  |  |
| Cerebellum 8^th^ Lobule | R/Vermis | 64 | †5.2 | | 10 | -60 | -32 |
| Parahippocampal G., Hippocampus | R | 13 | ‡4.88 | | 16 | -10 | -26 |
| Cerebellum 6^th^ Lobule | L | 151 | 4.49 | | -8 | -64 | -28 |
| Cerebellum 6^th^ Lobule | R | 62 | †3.84 | | 24 | -68 | -26 |
| *Persp. Deviant > Baseline* |  |  |  | |  |  |  |
| Superior Parietal C. | L | 86 | 4.36 | | -16 | -70 | 36 |
| † denotes regions significant at cluster level and FWE-corrected p < 0.05, after small volume correction for ROIs.  ‡ denotes significance at peak-level with FWE-corrected *p* < 0.05, after small volume correction for ROIs. | | | | | | | |
