# Supplementary figures and images for: Cerebellar‐hippocampal processing in passive perception of visuospatial change: An ego‐ and allocentric axis?
Source: Hum Brain Mapp. 2019 Nov 15;41(5):1153–66. doi: 10.1002/hbm.24865 (PMC7268078; doi:10.1002/hbm.24865)

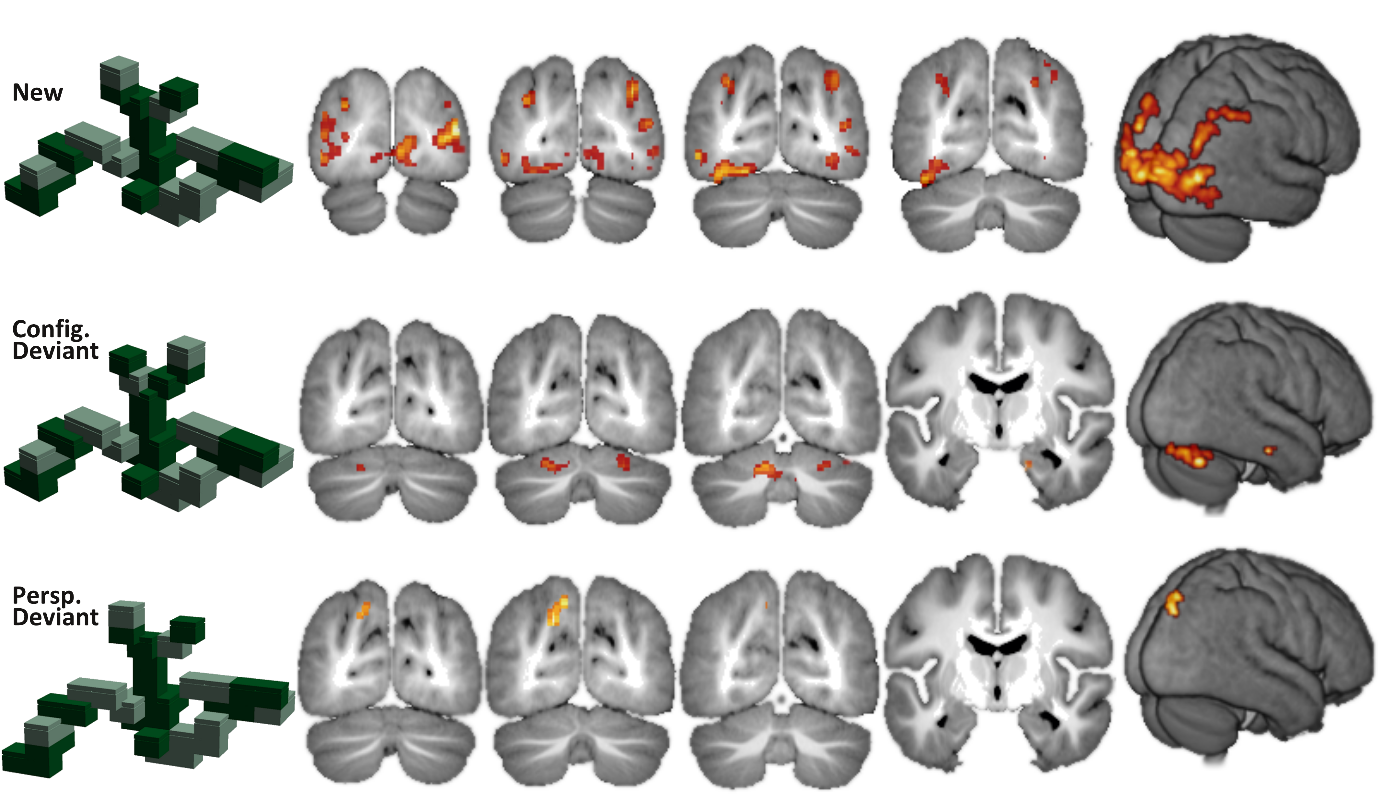

Supplement: Supplementary file 3 — Figure S1 Univariate results rendered onto mean anatomical image show visual cortical activity in response to new items, cerebellar and hippocampal activity for configurational (config.) deviants, and parietal activity for perspective (persp.) deviants. [file HBM-41-1153-s003.tif]

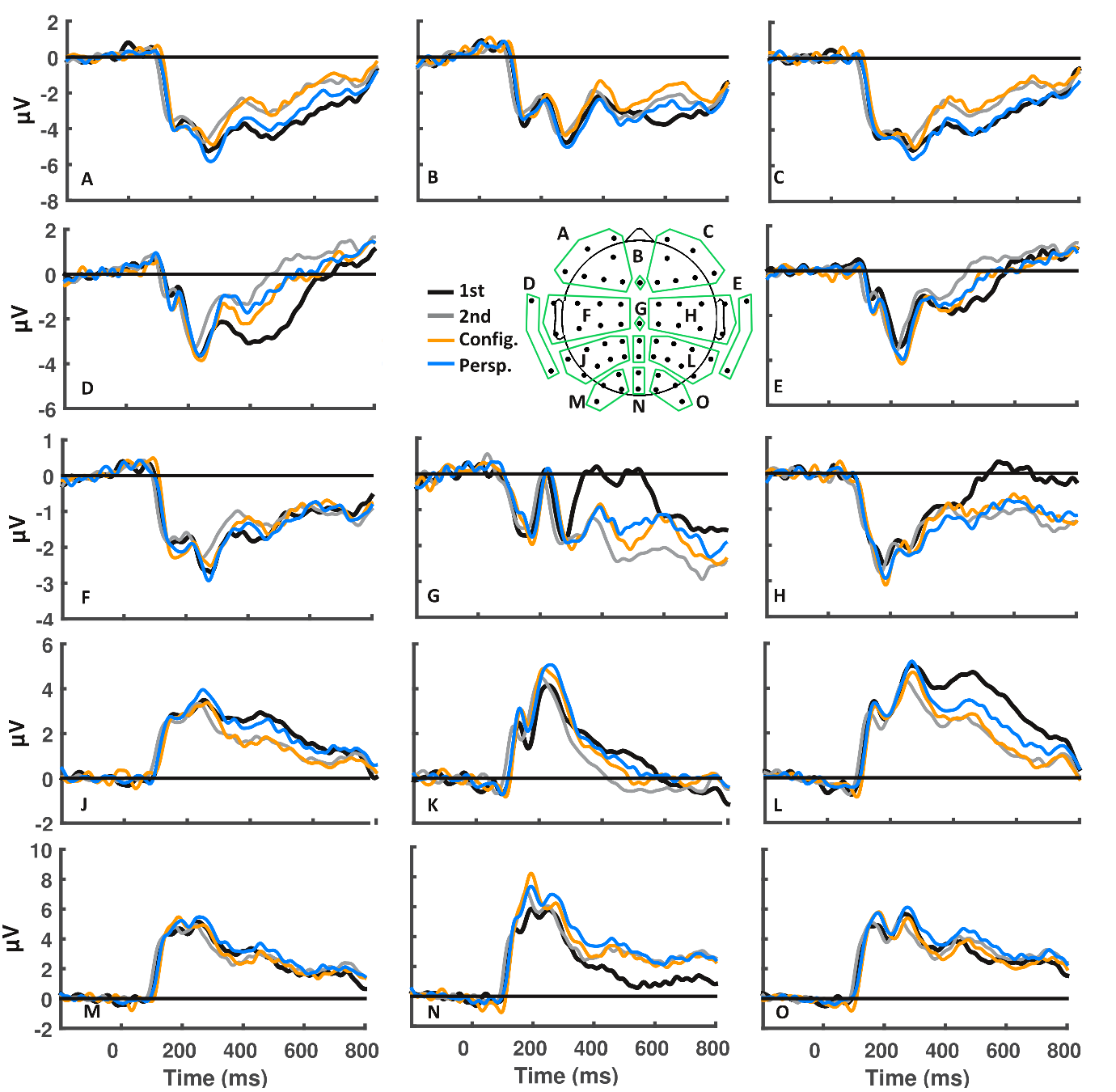

Supplement: Supplementary file 4 — Figure S2 Condition‐averaged ERPs across different electrode‐subsets. Conditions plotted are the for new and repeated standards (‘1st’ and ‘2nd’ presentations, respectively), as well as the configurational and perspective changes (‘Config.’ and ‘Persp.’). Time‐point 0 represents the onset of the respective image presentation. [file HBM-41-1153-s004.tif]

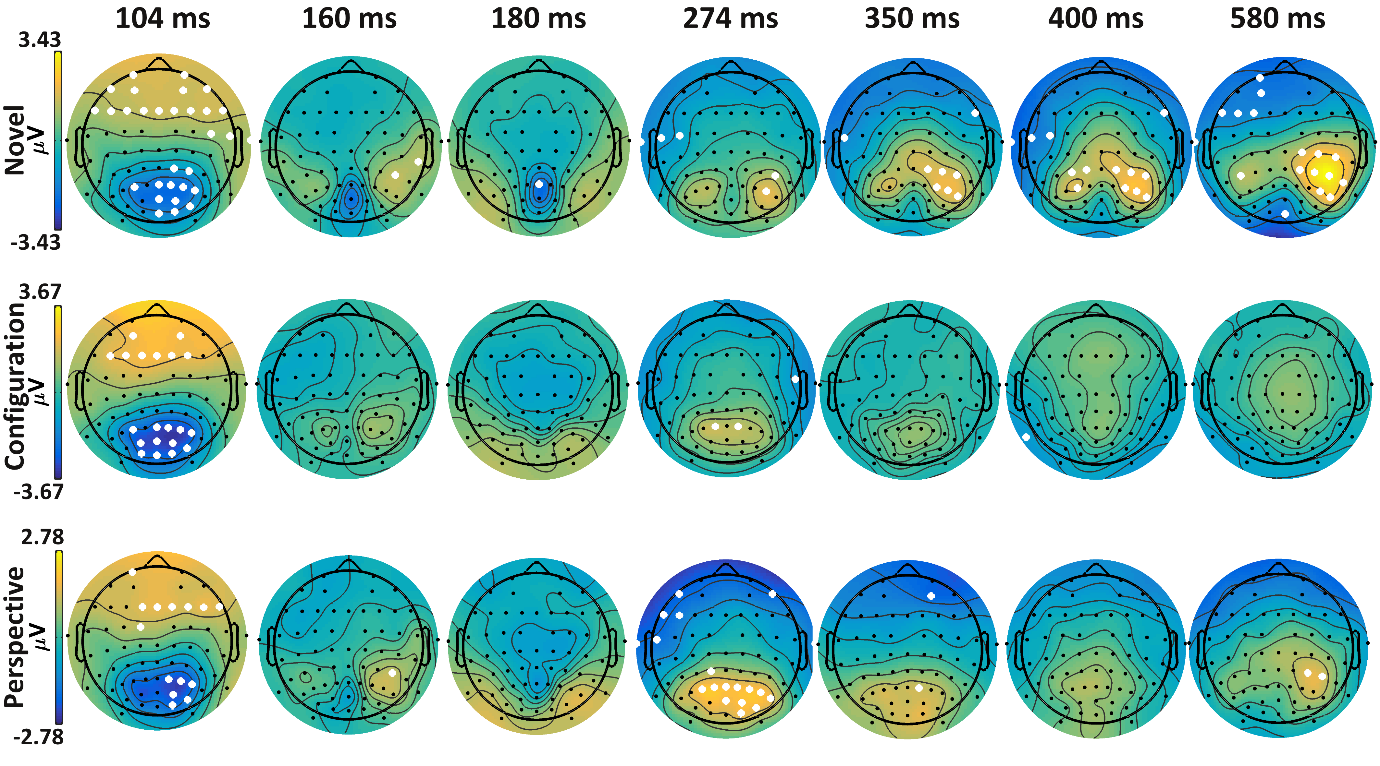

Supplement: Supplementary file 5 — Figure S3 ERP‐results show topographical differences between effects arising mostly in late time‐windows. Baseline‐subtracted topographies of time windows in which significant differences were found for novel (new) items, configurational deviants, or perspective deviants against baseline. White dots indicate electrodes significant at p < .05. [file HBM-41-1153-s005.tif]
